# Function and Regulation of Ammonium Transporters in Plants

**DOI:** 10.3390/ijms21103557

**Published:** 2020-05-18

**Authors:** Dong-Li Hao, Jin-Yan Zhou, Shun-Ying Yang, Wei Qi, Ke-Jun Yang, Yan-Hua Su

**Affiliations:** 1 State Key Laboratory of Soil and Sustainable Agriculture, Institute of Soil Science, Chinese Academy of Sciences, Nanjing 210008, China; dlhao@issas.ac.cn (D.-L.H.); 23240962@163.com (J.-Y.Z.); ysy@issas.ac.cn (S.-Y.Y.); 2 College of Resources and Environment, Shandong Agricultural University, Taian 271018, China; qiwei@sdau.edu.cn; 3 Agro-Tech Extension and Service Center, Zhucheng 262200, China; zcsyangkejun@163.com

**Keywords:** ammonium transporter, physiological roles, functional regulation, transport mechanism, yeast functional complementation, electrophysiology, genetic manipulation

## Abstract

Ammonium transporter (AMT)-mediated acquisition of ammonium nitrogen from soils is essential for the nitrogen demand of plants, especially for those plants growing in flooded or acidic soils where ammonium is dominant. Recent advances show that AMTs additionally participate in many other physiological processes such as transporting ammonium from symbiotic fungi to plants, transporting ammonium from roots to shoots, transferring ammonium in leaves and reproductive organs, or facilitating resistance to plant diseases via ammonium transport. Besides being a transporter, several AMTs are required for the root development upon ammonium exposure. To avoid the adverse effects of inadequate or excessive intake of ammonium nitrogen on plant growth and development, activities of AMTs are fine-tuned not only at the transcriptional level by the participation of at least four transcription factors, but also at protein level by phosphorylation, pH, endocytosis, and heterotrimerization. Despite these progresses, it is worth noting that stronger growth inhibition, not facilitation, unfortunately occurs when AMT overexpression lines are exposed to optimal or slightly excessive ammonium. This implies that a long road remains towards overcoming potential limiting factors and achieving AMT-facilitated yield increase to accomplish the goal of persistent yield increase under the present high nitrogen input mode in agriculture.

## 1. Introduction

Ammonium and nitrate, two inorganic nitrogen forms that plants can directly absorb from soils, are crucial for crop growth and yield formation. In dryland soils, nitrate serves as the main nitrogen. In flooded or acidic soils, ammonium becomes the major nitrogen form [1,2,3]. In agricultural production, application of nitrogen fertilizer can also lead to short-term ammonium dominance in soils, irrespective of soil type. Due to lower energy cost in the process of absorption and utilization than that of nitrate, ammonium is considered as a superior nitrogen source [4,5,6]. Concentration of ammonium nitrogen in soils is generally less than 1 mM [7,8]. In this concentration range, ammonium uptake in plants is dominated by the high-affinity ammonium absorption system, whose ability to absorb ammonium increases with the increase of supplied concentration and reaches saturation at approximately 1 mM. The relationship between the ammonium absorption rate and the supplied ammonium concentration conforms to the typical Michaelis–Menten equation [9,10,11]. Studies have shown that the molecular basis for the high-affinity ammonium uptake in plants is undertaken by the ammonium transporters (AMTs) [12,13,14,15,16]. Since ammonium/methylammonium possesses two forms, we refer different forms of these substances with following specific names. Ammonium or methylammonium represent the total amount of ionic and molecular formed substance. NH_4_^+^ and MeA^+^ represent ionic forms; NH_3_ and MeA represent molecular forms (Appendix A).

Structurally, an AMT consists of 11–12 transmembrane regions, with feature sequences “D (F Y W S) A G (G S C) X2 (L I V) (E H) X2 (G A S) (G A) X2 (G A S) (L F)” located at its transmembrane region 5 and “D D X (L I V M F C) (E D G A) (L I V AC) X3 H (G A L I V) X2 (G S) X (L I V A W) G” at transmembrane region 10 [12]. AMTs widely exist in plants and have large family members, which can be divided into two subtypes, AMT1 and AMT2, according to the distance of their genetic relationship [17,18]. Besides the above-mentioned process of acquisition of ammonium from soils, AMTs additionally engage diverse physiological roles in plants, and their activities are modulated not only at the transcriptional level, but also at the protein level. Classical AMT reviews that concentrated on the basic functionality and transcriptional regulations greatly facilitate our systemic understanding of this family, although they were published more than fifteen years ago [5,12,17]. Recently (2012), a review summarized the progress focused on the putative selective ammonium transport pathway of AMTs, which is structured by the ammonium binding/recruitment site, a so-called phenylalanine gate composed by two twin phenylalanine residues, a central section confined by two highly conserved histidines, and a cytoplasmic vestibule [16]. It facilitates our understanding of the microprocess of ammonium transport from the extracellular region to the cytosol by AMTs. As a supplement, this review highlights the recent progress made in understanding plant AMTs in terms of physiological function, transport mechanism, and regulatory mechanism, and it presents an outlook towards the future of this field of study.

## 2. The Physiological Roles of AMTs in Plants

### 2.1. AMT Mediates Ammonium Acquisition from Soil Solution

Screening of the cDNA library from model plant *Arabidopsis* using a yeast mutant (26972c) lacking its two ammonium transporter homologous genes, *Mep1* (methylammonium permease 1) and *Mep2* (methylammonium permease 2), led to the finding of the first ammonium transporter (AtAMT1;1) from the plant [19]. Expression of AtAMT1;1 enhances the growth of this yeast mutant under restricted ammonium supply and facilitates the energy-dependent active absorption of methylammonium, an analog of ammonium, suggesting a potential role of ammonium uptake by AtAMT1;1 [19]. Subsequent constructed yeast strain 31019b, which lacks its three Meps (Δmep-1, Δmep-2, Δmep-3), could not grow on the medium with <5 mM ammonium as the sole nitrogen source [20]. Expression of AtAMT1;1 in this yeast mutant can restore its growth under low ammonium conditions, further indicating that AtAMT1;1 is capable of ammonium uptake [21]. This 31019b yeast mutant hereafter is widely used for related yeast functional complementation due to its non-residual AMT homologous genes. When expressed in another heterologous expression oocyte system and investigated by two-electrode voltage clamp technique, AtAMT1;1 mediates ammonium influx with a *K_m_* value of 34 μM, supporting a high-affinity functional ammonium transporter [14,22,23]. In planta disruption of this root-expressed AtAMT1;1 results in about a 30% reduction of *Arabidopsis*’s ammonium absorption capacity, indicating that AtAMT1;1 does play a role in obtaining ammonium from soil solution in *Arabidopsis* [24,25]. The above-mentioned yeast functional complementation, oocyte two-electrode voltage clamp electrophysiology, and plant-level genetic manipulation (knockout/overexpression) form the three main methods used for functional verification/study of AMTs.

There are five AMTs in *Arabidopsis* roots, named AtAMT1;1, AtAMT1;2, AtAMT1;3, AtAMT1;5, and AtAMT2 [26,27]. Besides AtAMT1;1, the other four AMTs can also restore the growth of yeast mutant 31019b under restricted ammonium supply [21,26,28], indicating that these four AMTs are able to uptake ammonium. Electrophysiological results show that both AtAMT1;2 and AtAMT1;3 mediate high-affinity ammonium influx with *K_m_* values of ~100 μM [29,30]. In *Arabidopsis*, single knockout of root-expressed AtAMT1;2 or AtAMT1;3 leads to a 18%–26% or 30%–35% reduction of ammonium absorption, respectively [25,26], indicating that the two proteins also partly confer ammonium absorption along with AtAMT1;1 in planta. Double knockout of AtAMT1;1 and AtAMT1;2 resulted in a 60%–70% reduction [25], whereas knocking out all three of them (AtAMT1;1, AtAMT1;2 and AtAMT1;3) together resulted in a 90% reduction of the ammonium uptake capacity in *Arabidopsis*, allowing speculation that AtAMT1;5 is responsible for the remaining 10% [26]. On the one hand, these results strongly indicate that AMTs are the main driver of physiological ammonium absorption in plants (normally <1 mM). On the other hand, functional cooperation seemingly occurs amongst AtAMTs [26]. In terms of temporal and spatial organization, AtAMT1;1 and AtAMT1;3 are mainly expressed in the outer cell layers of roots and are responsible for absorbing ammonium from soil solution into the root cells and then transporting it through the symplasmic pathway. Ammonium that enters the intercellular space through the apoplastic pathway is obstructed by Casparian strip at endothelial cells. AtAMT1;2 is mainly expressed in endothelial cells, transporting this ammonium into the cytoplasm for symplasmic transport. AMT-mediated symplasmic and apoplastic ammonium transport pathways together contribute to the efficient acquisition of ammonium from soil solution [26,31].

Rice OsAMT1;1 restores the growth defect of the yeast mutant and mediates ammonium influx in oocytes [32], functioning in both cases like AtAMT1;1, indicating that OsAMT1;1 endows an ammonium uptake ability. Knockout of this root-expressed gene reduces ammonium uptake capacity of rice by about 25% [33], indicating that OsAMT1;1 contributes to the ammonium absorption of rice in vivo and suggesting that there may also be a functional cooperation between OsAMTs like that of AtAMTs. Additionally, many root-expressed functional AMTs have been identified, although only through one method among the three methods mentioned above. For example, overexpression of OsAMT1;1 in rice [34]; or NtAMT1.3 in tobacco [35]; or ZmAMT1.1a and ZmAMT1.3 [36], GhAMT1;3 [37], and PutAMT1;1 [38] in *Arabidopsis* can enhance the absorption of ammonium by plants under the condition of limiting ammonium supply, consequently increasing the biomass, which indicates that these proteins participate in obtaining ammonium from soil solution. The finding of either restoration of the growth of yeast mutants under restricted ammonium supply [1,26,39,40,41,42,43,44,45] or mediation of ammonium influx in oocytes in a heterologous system [46,47,48,49,50,51,52,53] suggests that these root-expressed AMTs potentially obtain ammonium nitrogen from soil solution. Given that temporal and spatial organization appears in AMTs from *Arabidopsis*, physiological roles of AMTs from other plant species with large family members should be determined by further mutant studies. The affinity constant of AMTs for ammonium varies, which may be beneficial for plants to obtain ammonium efficiently from the complex and changeable soil nutrient environment.

### 2.2. AMT Mediates Root-to-Shoot Transport of Ammonium

In model plant *Arabidopsis*, although the ammonium uptake capability of the root-expressed AtAMT2 is identified by yeast functional complementation [28,54], no substantial change of ammonium uptake capacity occurs upon the knockout or overexpression of this gene [26,55], suggesting that AtAMT2 alone does not play a significant role in ammonium acquisition from soil solution. Further study shows that the transcript abundances of AtAMT2 are preferentially concentrated to the pericycle upon sole ammonium exposure compared with the gene expression profile upon either the sole nitrate or the nitrogen-free treatment, suggesting that AtAMT2 may play a role in xylem ammonium loading. This notion is supported by the finding that knockout of AtAMT2 results in less translocation of ammonium to the shoots and reduced ammonium content in the xylem sap compared with that of the wild-type. Additionally, introduction of AtAMT2 on the background of either single AtAMT1;2 or AtAMT1;3 expression lines sharply facilitates the translocation of ammonium to shoots, indicating cooperation of AtAMT2 with AtAMT1;2, or AtAMT1;3 in the process of root-to-shoot ammonium translocation [56]. OsAMT1;1 is found in root stele, and knockout of this gene significantly reduces the root-to-shoot mobilization of ammonium, suggesting that OsAMT1;1 contributes to the root-to-shoot ammonium translocation [33]. OsAMT1;2 and ZmAMT1.3 are capable of ammonium acquisition when expressed in yeast mutant, and their transcripts are detected in the central cylinder [1,36], suggesting they are potentially involved in the root-to-shoot ammonium transport.

### 2.3. AMT Mediates Ammonium Transport in Leaves

The apoplast surrounding leaf cells contains substantial quantities of ammonium [57], which may derive from the xylem stream [58], from the photorespiratory cycle and protein catabolism, or directly from the atmosphere [59,60]. Both LeAMT1;2 and LeAMT1;3 are functional ammonium transporters, and their transcripts are found in tomato leaves. The peak transcript abundance of *LeAMT1;3* occurs during darkness, while that of *LeAMT1;2* appears at onset of light, suggesting that LeAMT1;3 may compensate for ammonium losses across the plasma membrane caused by de- and transamination processes, whereas LeAMT1;2 may be involved in the retrieval of photorespiration ammonium escaping from mitochondria and import of ammonium in the xylem [61]. The leaf-expressed *LjAMT1;1* and *BnAMT1;2* are conceived to play a similar role as that of *LeAMT1;2* [39,62]. *OsAMT1;1* [33], *NtAMT1.3* [35], *ZmAMT1.1a* [36], *ZmAMT1.3* [36], and *GhAMT1.3* [37] are all detected in the leaf. Amongst the three leaf types of young, mature, and senescent, transcript abundances of *PtrAMT1;6* and *PtrAMT3;1* are strongly induced in the senescent leaf. In addition to leaves, AMTs such as *PtrAMT2;2* and *PtrAMT1;1* are detected in petioles [40]. All these data suggest that the expression of these AMTs in leaf and petiole participates in the transport of ammonium nitrogen therein.

### 2.4. AMT Mediates Ammonium Acquisition in the Reproductive Organs

Transport of nitrogen to the developing flowers has a significant influence on flower set, pollen and embryo development, as well as seed production [63]. AtAMT1;4 is specifically expressed in pollen grains and pollen tubes of *Arabidopsis*, and its capability of ammonium uptake is confirmed by yeast functional complementation together with plant overexpression assays. Increasing methylammonium supply results in a persistent inhibition of the pollen germination rate of *Arabidopsis*, suggesting that the inflow of toxic methylammonium into the pollen occurs. AtAMT1;4 is speculated to mediate the entry of ammonium into pollen. However, no pollen phenotype appears in *Arabidopsis* after AtAMT1;4 is knocked out [27], which may be explained by the functional redundancy of pollen-tube-located AtAMT1;1 [64]. *AtAMT1;4* is solely expressed in male but not in female flower parts. In the flower organ of poplar, *PtrAMT1;5* is specific in the stamen and *PtrAMT1;6* in the female flower [40]. In sorghum flower organ, *SbAMT1;1*, *SbAMT1;2*, *SbAMT2;1*, *SbAMT3;1,* and *SbAMT3;3* are in pistils and stamens, whereas *SbAMT2;2* and *SbAMT3;2* are solely in pistils [65]. Expression of *LjAMT1;1-1;3* is also detected in flower [39]. Transcripts of *ZmAMT1.1a* are detected in the seeds [36]. These AMTs may play a role in providing ammonium nitrogen nutrition to the reproductive organs.

### 2.5. AMT Mediates Ammonium Transport from Symbiotic Fungi to Host Plants

More than 90% of plants form symbiotic relationship with fungi in soils. Symbiotic fungi can provide phosphate or nitrogen nutrients to their plant partners via mycorrhiza [66]. Ammonium is considered to be the main nitrogen form released by symbiotic fungi [67,68]. The expression of *SbAMT3;1* and *SbAMT4* genes in *Sorghum bicolor* is induced by mycorrhizal formation, and *SbAMT3;1* is restricted to cells containing developing arbuscules in the mycorrhizal roots [65]. Knockdown of *SbAMT3;1* reduces the rate of ammonium acquisition from symbiotic fungi and arrests the growth promotion effect experienced by plants after fungi inoculation [69]. These results confirm the role of AMTs in importing ammonium nitrogen from the symbiotic fungus to the plant partner. The *LjAMT2;2* from legume *Lotus japonicas* is exclusively expressed in the mycorrhizal roots and preferentially localized in the arbusculated cells; it is proposed to participate in the transfer of ammonium nitrogen provided by symbiotic fungi into plant cytoplasm. LjAMT2;2 functions as an acid-stimulated ammonium transporter, a property that is compatible with the acidic environment near the periarbuscular membrane. The periarbuscular membrane is the place where the final stage of ammonium transfer to the plant occurs [70]. Of the 16 *GmAMTs* in legume soybean, transcripts of at least 5 members are induced by arbuscular mycorrhiza, in which *GmAMT4;1* is mostly induced. GmAMT4;1 is a functional ammonium transporter, and its expression is restricted to the arbuscular cortex cells, mainly located in the branch domain of periarbuscular membranes, rather than the plasma membrane, suggesting the GmAMT4;1 facilitates the transfer of ammonium in the periarbuscular space into the cortex cells of soybean [71]. *LeAMT4* and *LeAMT5* from non-legume tomato are exclusively expressed in mycorrhizal roots, and phylogenetic tree analysis shows that they are orthologous with LjAMT2;2 [72]. Transcriptional abundances of *PttAMT1;2*, *PoptrAMT1.2b*, *PoptrAMT1.3*, *PoptrAMT1.4a*, and *PtrAMT1;2* from other non-legume poplars are also strongly induced by mycorrhizal formation. The ability of PttAMT1;2 and PtrAMT1;2 to uptake ammonium is identified by yeast functional complementation [40,73]. Therefore, all these mycorrhizal-formation-induced AMTs potentially participate in the transfer of ammonium at the plant–fungus interface to the plant host, in legumes and non-legumes.

### 2.6. AMT Is Required for Root Development

Unlike nitrate, which mainly controls the elongation of lateral roots, ammonium supply mainly induces the formation of lateral roots and the branching of higher-order lateral roots in *Arabidopsis*. This root development phenotype upon ammonium exposure is almost lost in the qko mutant, in which four AMTs (AtAMT1;1, AtAMT1;2, AtAMT1;3, and AtAMT2;1) are knocked out. Even when increasing the nitrogen content in qko mutant to levels that are comparable to those of the wild-type by providing ammonium at the millimolar level, the suppressed higher lateral root branching in qko mutant still cannot be restored, indicating that this case cannot be simply attributed to a nutrient issue, but rather to an ammonium-triggered signal response process that is related to AMTs. The inhibitory effect of third-order lateral root formation in qko can be only suppressed by AtAMT1;3, but not by other AMTs, indicating that in addition to being a transporter, AtAMT1;3 is required in the process of ammonium-triggered lateral root branching [74].

Increasing the supply concentration of ammonium from 1 to 10 mM (high ammonium) inhibits the root growth of *Lotus japonicas* and simultaneously results in an upregulation of *LjAMT1;3* transcripts in roots. When equivalently high nitrate is used as an alternative nitrogen source, this root growth inhibition phenotype disappears, suggesting that the phenotype (short root) induced by high ammonium is not simply caused by a nutrient issue, but most likely by a high-ammonium-triggered signal event. Overexpression of LjAMT1;3, but not LjAMT1;1, in *Lotus japonicas* reproduces this short-root phenotype, irrespective of external supplied ammonium concentration, indicating that the short root phenotype is LjAMT1;3-specific. These results together with its root stele expression pattern indicate that LjAMT1;3 is required for the short root phenotype upon high concentration of ammonium exposure [75,76].

In *Medicago truncatula*, the premature arbuscule degeneration (PAD) can be alleviated when phosphate transporter PT4 knockout mutant is nitrogen-deprived. Given that ammonium is the main nitrogen form transferring between symbiotic systems, it is speculated that the PAD may be related to AMTs therein. Although there are three *AMTs* (*LjAMT2;2, LjAMT2;3,* and *LjAMT2;4*) being strongly induced by mycorrhizal formation, knockout assays on the *pt4* mutant background show that LjAMT2;3, but not LjAMT2;4, is required for PAD suppression [77].

Although several AMTs are required for the root development, the mechanisms by which the AMTs sense the ammonium and regulate the root development remain less clear. Phytohormones are closely related to the root development, and ammonium triggers multi-phytohormone signal pathway mediated root development [78]. It is speculated that these AMTs are most likely linked to the ammonium-triggered phytohormone signal pathway.

### 2.7. The Role of AMT in Plant Disease Defense

Nitrogen status is closely related to plant disease defense [79,80]. In *Arabidopsis*, knockout of AtAMT1;1 enhances resistance to necrotrophic fungus *Plectosphaerella cucumerina* and reduces sensitivity to hemibiotrophic bacterium *Pseudomonas syringae*, indicating that AMTs may play a role in plant disease defense [81].

Infection with *Puccinia striiformis* f. sp. *tritici* reduces ammonium content in wheat leaves and induces gene expression of *TaAMT2;3a*. Knockdown of this gene significantly increases the ammonium content in the leaves and retards the growth of *P. striiformis*, decreasing the hyphal length and reducing the number of hyphal branches and haustorial mother cells, suggesting that TaAMT2;3a may play a role in transferring ammonium for the rust fungi and thus facilitate infection of wheat by stripe rust fungus [82].

When infected with stem rust disease pathogens, the transcript abundances of the three *TaAMTs* (*TaAMT1;1a*, *TaAMT1;1b*, and *TaAMT1;3*) are induced in susceptible varieties, but not in non-susceptible varieties of wheat. Additionally, the disease index of susceptible varieties is reduced along with the decrease of supplied ammonium concentration post-inoculation. These results, together with the capability of ammonium uptake in these three TaAMTs, suggest that ammonium influx mediated by TaAMTs may contribute to the infection of wheat stem rust disease [83]. AMTs may participate in plant–pathogen interaction by transport of ammonium.

Table 1 summarizes the physiological roles of plant AMTs.

## 3. Substrate Transport Mechanisms in AMTs

### 3.1. NH_4_^+^ Uniporter

In solution, there are permanently two forms of ammonium, ionic NH_4_^+^ and molecular NH_3_, which partially implies the diversity of the transport mechanisms in plant AMTs. LeAMT1;1 restores the growth of yeast mutants deficient in ammonium uptake under restricted ammonium supply, indicating that LeAMT1;1 is functional in ammonium uptake [61]. When expressed in oocytes, LeAMT1;1 mediates electrogenic ammonium influx, confirming that the substrate transported by LeAMT1;1 is in the charged form. The Hill coefficient obtained by the Michaelis–Menten equation fitting the relationship between the supplied ammonium and corresponding induced current is equal to 1, indicating that only one substrate binds to the binding site. The more negative the membrane potential, the smaller the *K_m_* value, suggesting that the substance transported across the membrane is in the cationic form. LeAMT1;1-mediated ammonium absorption is independent of external pH. The reversal potential of LeAMT1;1 moves toward positive direction only upon ammonium exposure, but not upon even a 1000-fold change of proton concentration. These data indicate that the electrogenic cationic substrate transported by LeAMT1;1 is NH_4_^+^, not H^+^. LeAMT1;1 functions as an NH_4_^+^ uniporter [46], which is further supported by the finding that each methylammonium ion transported by LeAMT1;1 carries a positive elementary charge [49]. A similar NH_4_^+^ uniport mechanism has been reported in AtAMT1;1 and OsAMT1;1 [14,32].

### 3.2. NH_3_/H^+^ Symporter

Similar to the above-mentioned results for LeAMT1;1 [49], each ^14^C-labeled methylammonium ion absorbed by TaAMT1;1 carries a positive charge, indicating that the substance transported across the membrane by TaAMT1;1 is NH_4_^+^ [50]. However, transport activity of this protein is stimulated by acid, allowing speculation that NH_4_^+^ is firstly perceived by the binding site and then transported through the tunnel of TaAMT1;1 in the form of divided NH_3_ and H^+^. Thus, in an acidic solution, H^+^ will more easily enter the cytoplasm due to a sharp increase in its concentration resulting in overall transport facilitation under acidic conditions. While in an alkaline solution, an opposite effect will occur. L56F mutation leads to the loss of acid stimulation in TaAMT1;1, which is mainly attributed to its promotion of H^+^ exiting from the tunnel. However, the issues of when and where H^+^ separates from NH_4_^+^, together with the identity of the pathway that is responsible for H^+^ transport, remain less clear [50].

Screening of mutations in AtAMT1;2 involving yeast survival on toxic methylammonium led to the finding of two unique mutants, Q67K and W145S, which are located far from its substrate transport tunnel. Evidenced by yeast functional complementation assay, Q67K and W145S mutants retain the capability of ammonium uptake. After being expressed in oocytes, they even show a greater plasma membrane location intensity than that of the AtAMT1;2 wild-type, and they substantially mediate the influx of ^15^N-labeled ammonium or ^14^C-labeled methylammonium. However, electrogenic transport of these substances occurring in the AtAMT1;2 wild-type disappears in the two mutants, suggesting the substance transported by the mutants is neither electrogenic NH_4_^+^ nor H^+^, but electroneutral NH_3_ molecules. The Q67K and W145S mutants achieve uncoupling of NH_3_ and H^+^ transport in AtAMT1;2, supporting that AtAMT1;2 functions as an NH_3_/H^+^ symporter [84].

### 3.3. NH_3_ Gas Channel

AtAMT2 restores the growth defect of yeast mutant 31019b under restricted ammonium, indicating its capability of ammonium uptake. When expressed in oocytes, AtAMT2 mediates ^14^C-labeled methylammonium, supporting that it functions normally in this system. However, non-electrogenic transport of either ammonium or methylammonium is detected in AtAMT2-expressing oocytes, suggesting that the substance transported by this protein is most likely the electroneutral NH_3_ molecule. Given that there is no significant growth difference shown in yeast harboring AtAMT2 at different pH values, the possibility of direct transport of NH_3_ is partially excluded. It is suggested that NH_4_^+^ is firstly recruited by the binding site and then transported across the membrane in the form of uncharged NH_3_ by dehydrogenation in AtAMT2 [28]. LjAMT2;2 shows a similar performance as that of AtAMT2 in both yeast mutant 31019b (restoration of growth defect of yeast mutant upon restricted ammonium) and oocyte (non-electrogenic transport of ammonium) systems, supporting an identical transport mechanism [70]. The two AMT2 members exhibit the same transport mechanism as their closely homologous EcAmtB, whose crystal structure is resolved [85,86,87].

### 3.4. NH_4_^+^/H^+^ Symporter

When expressed in oocytes, PvAMT1;1 mediates acid-stimulated electrogenic transport of ammonium. An equal proportion of NH_4_^+^ and H^+^ ions together confer the ideal slope of reversal potential (Er) responding to a 10-fold change of ion concentration, allowing speculation that the transport mechanism of this protein is NH_4_^+^/H^+^ symport. This notion is further supported by the cytoplasm acidification caused by ammonium exposure of PvAMT1;1. H211E mutation allowed both the acid-stimulated effect and cytoplasm acidification to disappear. Furthermore, the ideal slope of reversal potential (Er) responding to a 10-fold change of ion concentration in this mutant can be described by only one type of ion, NH_4_^+^. All these data suggest that PvAMT1;1 functions as an NH_4_^+^/H^+^ symporter and the amino acid H211 is required for the transport of coupled H^+^ [51].

Table 2 summarizes the four transport mechanisms in plant AMTs.

## 4. Functional Regulations of AMTs

### 4.1. Regulation by Transcription Factors

It is reported that transcripts of *AMTs* genes are detected not only at the vegetative growth stage, but also at the reproductive growth stage of plants. Apart from the constitutive expressions of several transcripts, most *AMTs* present temporal and spatial variations [88]. There are many factors involved in the transcriptional regulation of *AMTs*.The first factor is nitrogen status regulation. The gene expression of *AtAMT1;1-AtAMT1;3* [21,58], *AtAMT1;5* [26], *AtAMT2* [54] from *Arabidopsis*, *LeAMT1;1* [61] from tomato, *LjAMT1;1* and *LjAMT1;2* [39] from *Lotus japonicas*, *PtrAMT1;2* [40] from poplar, and *MtAMT1;1* [42] from *Medicago truncatula* are all induced by nitrogen starvation. However, under nitrogen starvation, gene expression of *BnAMT1;2* is unaffected [62] and transcript abundances of *ZmAMT1;1a* and *ZmAMT1;3* are downregulated [36]. Upon high nitrogen supply, the expression levels of *MtAMT1;4* and *MtAMT2;1* are significantly upregulated [42]. The transcripts of *BnAMT1;2* are enhanced as the amount of ammonium supplied is increased, but are downregulated under persisting high nitrogen [62]. The transcript abundances of *ZmAMT1;1a* and *ZmAMT1;3* are rapidly increased by resupply of ammonium to nitrogen-deprived maize seedlings, and their transcript levels are unchanged against the increase of supplied ammonium concentration. The triggered transcripts of *ZmAMT1;1a* and *ZmAMT1;3* only occur upon ammonium exposure, but not upon nitrate exposure or nitrogen-starvation. The upregulation occurring upon ammonium exposure in two *ZmAMT1* genes is independent of nitrogen status in maize [36]. The second factor is cytosolic glutamine (Gln) regulation. Transcripts of *AtAMT1;1*-*AtAMT1;3* [21,58,89] and *OsAMT1;3* [90] are suppressed by Gln, whereas those of *OsAMT1;1* and *OsAMT1;2* are Gln-induced [90]. The third factor is circadian rhythm or photoperiod regulation. Under a day/night cycle, transcripts of *LeAMT1;2* achieve the highest level under dark condition, while those of *LeAMT1;3* reach peak abundance at the beginning of light [61]. Diurnal rhythms have been also reported in *AMTs* from rice [34] and poplar [40]. The fourth factor is mycorrhizal formation regulation. Transcriptional upregulation of *AMTs* from various species is found upon mycorrhizal formation [40,65,70,71,73]. The fifth factor is CO_2_ regulation. The transcripts of *LeAMT1;2* and *LeAMT1;3* in leaves are almost unaffected by increasing CO_2_ concentration in atmosphere [61], whereas those of *AtAMT2* and *LjAMT1;1* are suppressed [39,55]. The sixth factor is leaf age regulation. Shoot-specific *PtrAMT1;6* and *PtrAMT3;1* are strongly upregulated during senescence [40].

The transcriptional regulations of *AMTs* are executed by serial transcription factors. It is reported that transcription factor indeterminate domain 10 (OsIDD10) binds to a cis-element motif present in the promoter region of *OsAMT1;2*, thus specifically activating its gene expression. In spite of reduction in transcript abundance, the effect of its stimulation by ammonium presence still remains in OsIDD10 knockout mutant, suggesting that additional transcription factors confer its transcription [91]. Subsequent findings show that another transcription factor HY5 (Long Hypocotyles 5) is able to negatively regulate the gene expression of *AtAMT1;2*, an orthologous genes of *OsAMT1;2* [92], whereas DNA binding with one finger (DOF) transcription factor *OsDOF18* positively modulates the transcript abundance of *OsAMT1;1*, *OsAMT1;3*, *OsAMT2;1,* and *OsAMT4;1* [93]. Additionally, the transcription factor *OsDof25* from rice is capable of positively regulating the transcripts of *AtAMT1;1* and *AtAMT2;1* from *Arabidopsis* [94].

### 4.2. Regulation by pH

To date, only transport activities of TaAMT1;1 and PvAMT1;1 are pH-dependent, exhibiting an acid-stimulated regulatory mode [50,51], whereas the transport activities of other AMTs such as LeAMT1;1 [46], LeAMT1;2 [47], AtAMT1;1 [14,22], and OsAMT1;1 [32] are pH-independent when expressed in oocytes. This information suggests diverse pH regulation modes in AMTs. Further mutation of amino acid site L56F located in TaAMT1;1 or H211E in PvAMT1;1 leads to a pH-insensitive case [50,51]. It is worth noting that the two residues are highly conserved among all above-mentioned AMTs [16], irrespective of their pH regulatory mode, implying that pH regulation of AMTs may be determined by a multi-site network with the participation of L56 and H211.

### 4.3. Regulation by Phosphorylation

In AtAMT1;1, substitution of the carboxyl-terminal-located residue T460 with A, to mimic the de-phosphorylated status of this site, leads to an active ammonium transporter. However, substitution of T460 with D, to mimic its phosphorylated status, results in an inactive transporter. These results suggest that the phosphorylation status of T460 is essential for the functional switch of AtAMT1;1 [95]. Equivalent T460 residue mutations of either AtAMT1;2 or AtAMT1;3 result in similar effects [29,30,96], indicating that this mode of phosphorylation regulation may be of universal significance in AMTs. In *Arabidopsis*, apoplastic ammonium is proposed to act as a signaling molecule to induce T460 phosphorylation in a time- and concentration-dependent manner. Reduction of ammonium uptake appears immediately in planta after the T460 is phosphorylated, in agreement with its performance in a heterologous system. Given that overaccumulation of ammonium in plants is toxic [97,98,99], the rapid shut-off of AtAMT1;1 by T460 phosphorylation is conceived to be important for preventing ammonium toxicity [100]. However, the protein kinase that potentially exerted the phosphorylation was unclear then. FK506 is an inhibitor of a calcium-regulated serine/threonine-specific protein phosphatase [101,102], whereas genistein is a potent inhibitor of tyrosine-specific protein kinases [103,104]. Introduction of either FK506 or genistein suppress the ammonium uptake by magnitude of at least 30% in OsAMT1;1, suggesting that the AMTs may be regulated by multiple phosphorylation pathways [32]. The protein kinase CIPK23 has been shown to phosphorylate the corresponding T460 residue in AtAMT1;1 and AtAMT1;2, in a CBL1-dependent manner. However, substantial T460 phosphorylation signals in these two proteins, although with reduced levels, are still detected in *cipk23* mutant, further suggesting that there are extra protein kinases or phosphatases involved in equivalent T460 phosphorylation of AMTs, except for CIPK23 [105]. Subsequent study showed that another protein kinase, OsACTPK1, is able to mediate the phosphorylation of equivalent T460 in OsAMT1;2. Under ammonium-deprived condition, the transcript abundance of OsACTPK1 is poor, thus indicating that the non-phosphorylated OsAMT1;2 is in its active status in order to acquire ammonium for growth. Upon the increasing supply of the ammonium concentration, the transcript abundance of OsACTPK1 sharply increases and turns OsAMT1;2 into an inactive ammonium transporter by phosphorylation of its equivalent T460 site, preventing ammonium toxicity [106]. It is worth noting that, in addition to the equivalent T460, phosphorylation signals are also detected at seven sites (S475, S488, S490, S492, T496, T497) in AtAMT1;1 and at three sites (S480, S487, T494) in AtAMT1;3 [99,107,108,109]. Resupply of nitrate, but not ammonium, to nitrogen-deprived seedlings results in a rapid de-phosphorylation of the T494 site in AtAMT1;3, a status which is associated with an enhanced transport activity, consequently contributing to the promotion of overall ammonium uptake in *Arabidopsis*. Here, occurrence of the de-phosphorylation of the T494 site is seemingly associated with a phosphatase, rather than a protein kinase. The regulation of T494 is independent of the equivalent T460 [30]. Taken together, the phosphorylation regulation of AMTs may be involved in multi-site phosphorylation events launched by complex nitrogen signals, with the participation of numerous plant kinases/phosphatases.

### 4.4. Regulation by Internalization and Heterotrimerization

Under nitrogen-deprived condition, AtAMT1;3 mainly targets the plasma membrane. Upon high ammonium exposure, the quantities of the plasma-membrane-located AtAMT1;3 protein are decreased through clustering and endocytosis into the cytoplasm, partially preventing ammonium toxicity. The internalization of AtAMT1;3 occurs mainly through clathrin-mediated endocytic pathways [110].

Forming of a trimer is required for a functional AMT [111]. Co-expression of the inactive G458D-LeAMT1;1 mutant with either LeAMT1;1 or LeAMT1;2 wild-type in oocytes results in a functional inhibition of the latter protein, suggesting that heterotrimerization occurs between them [47]. It is reported that AtAMT1;1 not only forms a homotrimer, but also forms a heterotrimer with AtAMT1;3. Substitution of the equivalent T460 site with D to mimic its phosphorylated status in these two proteins results in the loss of function. Overexpression of AtAMT1;3TD on the background of lines only harboring AtAMT1;3 leads to a decrease in ammonium uptake, while overexpression of AtAMT1;3TD on the background of lines only harboring AtAMT1;1 performs a comparable inhibitory effect, suggesting that AtAMT1;3TD possesses a similar preference to assemble with AtAMT1;1 or AtAMT1;3. Apoplastic ammonium-triggered phosphorylation of the equivalent T460 site is only detected in AtAMT1;1, but not in AtAMT1;3. Considering that once a single monomer among the homotrimer or the heterotrimerization is phosphorylated, the overall AMTs are inactive, the heterotrimer formed by AtAMT1;1 and AtAMT1;3 may facilitate the sensing of ammonium signal, thus achieving rapid shut-off of AtAMT1;3 at toxic ammonium concentration [95].

**Figure 1:**
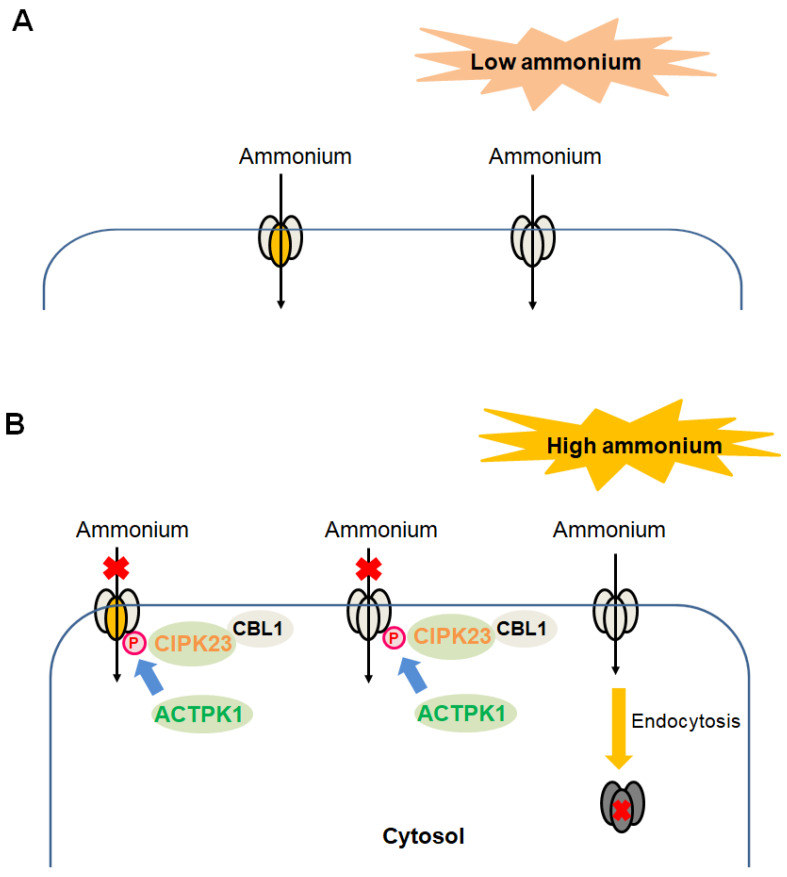
Functional regulations of plant AMTs at the protein level in cells. (**A**,**B**), Regulatory strategies used by plant AMTs upon either the exposure to low concentrations of ammonium (**A**) or the high concentrations of ammonium (**B**). When subjected to low concentrations of ammonium, AMTs in the homotrimer form (same-colored ellipses) or the heterotrimerization form (different-colored ellipses) are active ammonium transporters, mediating the uptake of ammonium into the cytosol. When exposed to high concentrations of ammonium, the CBL1–CIPK23 protein kinase complex as well as the protein kinase ACTPK1 phosphorylates the carboxyl terminus of AMTs, resulting in functional shut-off. Once a single monomer among the homotrimer (same-colored ellipses) or the heterotrimerization (different-colored ellipses) is phosphorylated, the AMT is inactive overall. Additionally, the quantities of plasma-membrane-located AMTs are decreased through clustering and endocytosis into the cytoplasm. Both cases reduce the ammonium influx across the plasma membrane, preventing ammonium toxicity. However, upon shifting of plant cells from high ammonium exposure to the low ammonium condition that requires AMTs to exert ammonium influx, the processes for the de-phosphorylation (by phosphatase) of AMTs to re-enable them as functional ammonium transporters and for the re-trafficking of AMTs to the plasma membrane to facilitate transport remain to be determined.

Figure 1 summarizes the functional regulation of plant AMTs at the protein level.

## 5. Conclusion and Prospect

In flooded or acidic soils, ammonium serves as the main nitrogen form available for plant growth. This is also the case for soil conditions shortly after nitrogen fertilizer application in agricultural practice, irrespective of soil type. Ammonium is additionally the major nitrogen form released by fungi at the plant–fungus interface and provided to the host plant. Besides the acquisition of ammonium from these resources, AMTs are also involved in serial physiological processes, including transferring ammonium from root to shoot, ammonium transfer in reproductive organs, and transporting ammonium for disease defense. Besides being a transporter, several AMTs are required for modification of root structure through extra- or intercellular perception of the ammonium signal. Sophisticated regulatory strategies are assigned to AMTs at transcriptional and protein levels, protecting plant growth from harm due to insufficient or excessive acquisition of ammonium. The physiological role of an AMT cannot be replaced by another one, even if it has similar transport property. Based on the progress that has been made in determining the function and regulation of AMTs, as summarized in this review, we believe that the following aspects still need further consideration. (i) Studies are presently mainly focused on the AMT1 subfamily, and research on the AMT2 subfamily needs to be strengthened. Except for the case of *Arabidopsis*, which contains only one AMT2, the number of members of AMT2 in many species far exceeds that of AMT1 [18,40,65,71,112,113]. For example, there are nine AMT2, but only three AMT1 members in the rice genome [18]. Together with its low homology to AMT1, AMT2 may play a different role from that of AMT1. (ii) Understanding of the pathways of functional regulation and their physiological significance needs to be strengthened. For example, phosphorylation and de-phosphorylation alternatively occur in AMTs, depending on the supplied ammonium conditions. However, present research is mainly focused on the protein-kinase-triggered phosphorylation event, whereas there are no reports on the phosphatase-dominated de-phosphorylation cases. (iii) Understanding of the physiological role of AMTs in specialized organs needs to be further strengthened. The dominant role of AMTs in ammonium acquisition from soils has been clearly established. However, although results have shown that AMTs located in other organs, such as leaves, petioles, flowers, and fruits, potentially play a role in ammonium acquisition, the contribution ratio of these AMTs to the overall ammonium nitrogen transport remains unclear. In addition to AMTs, the potassium channel, aquaporin (AQP), and non-selective cation channel (NSCC) are conceived to transport ammonium, although working at a higher concentration than AMTs do. (iv) Present studies mainly focus on the plasma-membrane-located AMTs, with less attention paid to AMTs potentially located in other organelles. (v) Genetic manipulations of AMTs aiming to increase the biomass or yield are not very satisfactory [114,115,116,117]. Although overexpression of AMTs enhances plant growth under nitrogen-insufficient condition, strong growth suppression, not facilitation, unfortunately occurs when they are exposed to optimal or excessive ammonium supply [34]. Therefore, overcoming the limiting factors that hinder the exertion of their positive effects is the core pursuit of AMT research, to achieve the goal of continuous yield increase under the present high nitrogen input mode in agricultural production.

## Figures and Tables

**Table 1:** Physiological roles of ammonium transporters (AMTs) in plants.

Protein Name	Location	Evidenced by Plant KO/OE Lines	Physiological Roles	References
			**Ammonium uptake from soils**	
AtAMT1;1	Root, rhizodermis, and root hairs	Yes	Symplastic transport of ammonium, accounts for 30%–35% of total ammonium uptake in roots	[19,21,22,23,24,25,26]
AtAMT1;2	Root, endothelial cells	Yes	Apoplastic transport of ammonium, accounts for 18%–26% of total ammonium influx in roots	[21,26,29]
AtAMT1;3	Root, rhizodermis, and root hairs	Yes	Symplastic transport of ammonium, accounts for 30%–35% of total ammonium uptake in roots	[21,25,26,30]
AtAMT1;5	Root, rhizodermis, and root hairs	No	Potential ammonium uptake in roots	[26]
OsAMT1;1	Root, epidermis	Yes	Contributes 25% of ammonium uptake in roots	[32,33,34]
NtAMT1.3	Root	Yes	Ammonium uptake in roots	[35]
ZmAMT1.1a	Root, epidermal cells	Yes	Ammonium uptake in roots	[36]
ZmAMT1.3	Root, epidermal cells	Yes	Ammonium uptake in roots	[36]
GhAMT1;3	Root	Yes	Ammonium uptake in roots	[37]
PutAMT1;1	Root	Yes	Ammonium uptake in roots	[38]
OsAMT1;2	Root, exodermis cells	No	Potential ammonium uptake in roots	[1]
LjAMT1;1-1;3	Root	No	Potential ammonium uptake in roots	[39]
PtrAMT1;2	Root	No	Potential ammonium uptake in roots	[40]
LeAMT1;1	Root	No	Potential ammonium uptake in roots	[41,46,49]
MtAMT1;1, MtAMT2;1	Root, rhizodermal cells	No	Potential ammonium uptake in roots	[42]
PpAMT1.3, PpAMT2;3	Root	No	Potential ammonium uptake in roots	[43]
OsAMT1;3	Root	Yes	Potential ammonium uptake in roots	[44]
PbAMT1;3	Root	No	Potential ammonium uptake in roots	[45]
LeAMT1;2	Root	No	Potential ammonium uptake in roots	[47,48]
TaAMT1.1	Root	No	Potential ammonium uptake in roots	[50]
PvAMT1;1	Root	No	Potential ammonium uptake in roots	[51]
			**Root-to-shoot translocation**	
AtAMT2	Root, pericycle	Yes	Ammonium root-to-shoot translocation	[56]
OsAMT1;1	Root, stele	Yes	Ammonium root-to-shoot translocation	[32,33,34]
ZmAMT1.3	Root, pericycle cell layer	No	Ammonium root-to-shoot translocation	[36]
OsAMT1;2	Root, pericycle cells	No	Ammonium root-to-shoot translocation	[1]
			**Ammonium transport in leaves**	
LeAMT1;2	Leaf	No	Potential retrieval of photorespiration ammonium escaping from mitochondria and import of ammonium in the xylem	[61]
LeAMT1;3	Leaf	No	Potential compensation for ammonium losses across the plasma membrane caused by de- and transamination processes	[61]
LjAMT1;1	Leaf	No	Potential retrieval of photorespiration ammonium escaping from mitochondria and import of ammonium in the xylem	[39]
BnAMT1;2	Leaf	No	Potential retrieval of photorespiration ammonium escaping from mitochondria and import of ammonium in the xylem	[62]
PtrAMT1;6, PtrAMT3;1	Senescent leaf	No	Potential ammonium transport in leaf	[40]
PtrAMT2;2, PtrAMT1;1	Petioles	No	Potential ammonium transport in leaf	[40]
OsAMT1;1	Leaf mesophyll cells	No	Potential ammonium transport in leaf	[32,33,34]
NtAMT1.3	Leaf	Yes	Ammonium transport in leaf	[35]
ZmAMT1.1a	Leaf	No	Potential ammonium transport in leaf	[36]
ZmAMT1.3	Leaf	No	Potential ammonium transport in leaf	[36]
GhAMT1;3	Leaf	No	Potential ammonium transport in leaf	[37]
			**Ammonium acquisition in the reproductive organs**	
AtAMT1;4	Flower	Yes	Ammonium acquisition in flower	[27]
AtAMT1;1	Flower	No	Potential ammonium acquisition in flower	[64]
PtrAMT1;5	Stamen	No	Potential ammonium acquisition in flower	[40]
PtrAMT1;6	Female flower	No	Potential ammonium acquisition in flower	[40]
SbAMT1;1, SbAMT1;2, SbAMT2;1, SbAMT3;1, and SbAMT3;3	Pistils and stamens	No	Potential ammonium acquisition in flower	[65]
SbAMT2;2 and SbAMT3;2	Pistils	No	Potential ammonium acquisition in flower	[65]
LjAMT1;1-1;3	Flower	No	Potential ammonium acquisition in flower	[39]
ZmAMT1.1a	Seeds	No	Potential ammonium acquisition in seeds	[36]
PutAMT1;1	Anther	No	Potential ammonium acquisition in flower	[38]
			**Ammonium transport from symbiotic fungi to host plants**	
SbAMT3;1	Cortex cells containing developing arbuscules	Yes	Transferring ammonium to host plant	[65,69]
LjAMT2;2	Mycorrhizal roots, arbusculated cells	No	Potentially transferring ammonium to host plant	[70]
GmAMT4;1	Arbuscular cortex cells	No	Potentially transferring ammonium to host plant	[71]
LeAMT4, LeAMT5	Mycorrhizal roots	No	Potentially transferring ammonium to host plant	[72]
*PttAMT1;2*	Mycorrhizal roots	No	Potentially transferring ammonium to host plant	[73]
*PoptrAMT1.2b*, *PoptrAMT1.3*, *PoptrAMT1.4a*, and *PtrAMT1;2*	Mycorrhizal roots	No	Potentially transferring ammonium to host plant	[40,73]
			**Required for root development**	
AtAMT1;3	Root	Yes	Required for high-order lateral root branching upon ammonium exposure	[74]
LjAMT1;3	Root	Yes	Required for short root phenotype upon high concentration of ammonium exposure	[75,76]
LjAMT2;3	Mycorrhizal roots	Yes	Required for root premature arbuscule degeneration suppression	[77]
			**Roles in plant disease defense**	
AtAMT1;1	Root	Yes	Enhances resistance to necrotrophic fungus *Plectosphaerella cucumerina* and reduces sensitivity to hemibiotrophic bacterium *Pseudomonas syringae*	[81]
TaAMT2;3a	Leaf	Yes	Retards the growth of *P. striiformis*	[82]
TaAMT1;1a, TaAMT1;1b, and TaAMT1;3	Leaf	No	Participates in plant–pathogen interaction by transport of ammonium	[83]

Note: KO, knockout; OE, over-expression.

**Table 2:** Transport mechanisms of plant AMTs.

Protein Name	Transport Mechanisms	Supporting Evidence	References
	**NH_4_^+^ uniport**		
LeAMT1;1	NH_4_^+^ uniport	(i) Electrogenic transport of ammonium. (ii) The more negative the membrane potential, the smaller the *K_m_* value, suggesting the cationic transport. (iii) Reversal potential moves towards positive direction only by ammonium introduction. (iv) pH-independent. (v) Each methylammonium ion transported carries a positive elementary charge.	[46,49]
AtAMT1;1	NH_4_^+^ uniport	(i) Electrogenic transport of ammonium.(ii) pH-independent.	[14]
OsAMT1;1	NH_4_^+^ uniport	(i) Electrogenic transport of ammonium. (ii) pH-independent.	[32]
	**NH_3_/H^+^ cotransport**		
TaAMT1;1	NH_3_/H^+^ cotransport	(i) Electrogenic transport of ammonium. (ii) Each methylammonium ion transported carries a positive elementary charge. (iii) Stimulated by acidic pH.	[50]
AtAMT1;2	NH_3_/H^+^ cotransport	(i) Electrogenic transport of ammonium. (ii) Mutation of Q67H and W145S results in uncoupling transport of NH_3_ and H^+^.	[84]
	**NH_3_ cotransport**		
AtAMT2	NH_3_ cotransport	(i) Electroneutral transport of ammonium. (ii) pH-independent.	[28]
LjAMT2;2	NH_3_ cotransport	(i) Electroneutral transport of ammonium. (ii) Stimulated by acidic pH.	[70]
	**NH_4_^+^/H^+^ cotransport**		
PvAMT1;1	NH_4_^+^/H^+^ cotransport	(i) Electrogenic transport of ammonium. (ii) NH_4_^+^ and H^+^ ions together describe the ideal slope of reversal potential changes against 10-fold substrate concentration changes, and H199E mutation causes only NH_4_^+^ to describe the ideal slope. (iii) Cytosol acidification by PvAMT1;1 upon ammonium exposure, but this is not the case by H199E mutation.	[51]

## Appendix A

Ammonium or methylammonium represent the total amount of ionic and molecular formed substance. NH_4_^+^ and MeA^+^ represent ionic forms; NH_3_ and MeA represent molecular forms.
